# Impact of social determinants of health on individuals living with generalized myasthenia gravis and implications for patient support programs

**DOI:** 10.3389/fpubh.2023.1147489

**Published:** 2023-05-19

**Authors:** Tom Hughes, Ashley E. L. Anderson, Ali A. Habib, Kathy Perez, Cathleen Bergin, Sharon Suchotliff, Cecilia Zvosec, Dajzsa McDaniel, Mai Sato, Albert Whangbo, Glenn Phillips

**Affiliations:** ^1^argenx US Inc., Boston, MA, United States; ^2^Department of Neurology, Houston Methodist, Houston, TX, United States; ^3^UCI Health ALS & Neuromuscular Center, University of California Irvine, Orange, CA, United States; ^4^ZS Associates, New York, NY, United States

**Keywords:** patients, caregivers, myasthenia gravis, social determinants of health, burden, patient support services, mixed methods

## Abstract

**Introduction:**

Social determinants of health (SDOH) are important contributors to health outcomes, and better understanding their impact on individuals diagnosed with rare, chronic diseases with high burden and unmet need is critical. Characterizing SDOH burden can help improve the design of patient support programs (PSPs), using targeted approaches to remove barriers to access.

**Methods:**

This study used a mixed-methods strategy employing a quantitative survey, which was designed based on qualitative interviews, to understand the unmet needs and awareness/utilization of PSPs among individuals living with generalized myasthenia gravis (gMG) and experiencing SDOH barriers. The survey was completed by 38 individuals living with gMG, of which the majority were non-White/Caucasian, unemployed, low income, and enrolled in public insurance. Common SDOH challenges, awareness/utilization of available PSPs, and unmet needs were identified.

**Results:**

Financial and mental health concerns were the most common among individuals living with gMG and experiencing SDOH barriers throughout diagnosis, accessing treatment, initiating treatment, and continuing treatment. Awareness and utilization of existing support services were low, especially when accessing treatment. Educational, financial, and personalized support with high “human touch” were commonly perceived as the most valuable resources.

**Implications:**

To better serve the needs of individuals with gMG experiencing SDOH barriers, PSPs should use a targeted approach to offer services tailored to harder-to-reach populations. Further, providers, advocacy groups, manufacturers, and public organizations in the gMG ecosystem should strengthen collaborations with PSPs to enable individuals living with gMG to access the services they need to improve their health outcomes.

## 1. Introduction

Fluctuating and debilitating muscle weakness involving facial, bulbar, cervical, axial, and limb muscles is the hallmark of generalized myasthenia gravis (gMG)—a rare, autoimmune, chronic neuromuscular junction disorder (1–4). gMG affects more than 50,000 individuals in the United States and is more commonly diagnosed in women <40 years and men >60 years of age (5, 6). Although symptomatic immunosuppressive medications used as standard-of-care treatments in the management of gMG can be effective (7), individuals living with gMG continue to experience a high burden of disease (8, 9), with up to 20% experiencing a myasthenic crisis requiring intensive care and respiratory support over their lifetime (10). Impairment of daily activities including eating, driving, walking, and housework due to clinical symptoms of gMG (3, 11) can be compounded by common comorbidities such as hypertension and diabetes (12). Moreover, mood disorders, which can lead to clinical worsening of gMG, affect 41% of diagnosed individuals (13), contributing to low health-related quality of life (HRQoL) (5, 11, 14, 15). Substantial economic burden is attributed to high annual medical costs, challenges in maintaining employment, and approximately one-third of diagnosed individuals requiring frequent care from their primary caregiver (1, 3, 11, 16–19). While multiple recently launched biologic treatment options offer hope for improved outcomes (20–24), the aforementioned challenges still remain in gMG management as the landscape of care continues to evolve (25).

Critically, a major knowledge gap exists in understanding the burden and impact of social determinants of health (SDOH), defined as nonmedical, nonbiological, and nongenetic factors of the environment or individuals’ lives that affect health, functioning, and HRQoL outcomes—including conditions in which people are born, grow, work, live, and age (26, 27)—in gMG. SDOH has emerged as a key consideration across healthcare as SDOH barriers can be associated with increased health risks and implicit bias that can affect patient–provider interactions and patient health outcomes (28–32). Age, employment, and education status were shown to affect HRQoL in gMG in Germany and Poland (14, 33), and both treatment utilization and clinical outcomes significantly differed by race among individuals who were hospitalized for gMG in the United States (34). While SDOH-based variances have been reported around clinical phenotypes of gMG (35–37), further evidence demonstrating the impact of SDOH on day-to-day experiences in gMG is limited.

Better understanding the impact of SDOH on individuals’ support needs is vital for gMG support networks to reach those who may benefit the most. In gMG, support is commonly offered through patient advocacy groups (PAGs), which are nonprofit organizations that offer a variety of resources including, but not limited to, support groups, educational events, and health and wellness resources for patients throughout the diagnostic journey and beyond. PAGs can be regional or national, with a broad range of scope including representing the patient voice with policymakers, promoting the needs of patients and families, and raising awareness of the condition to the general public. Patient support in gMG is also offered through patient support programs (PSPs), often sponsored by providers, pharmacy benefit managers, payers, PAGs, or pharmaceutical companies. Offering interventions that aim to improve access, usage, and adherence to treatments and disease self-management, PSPs can have a positive impact on clinical, humanistic, and economic outcomes (38–40), especially when designed to match the needs of individuals who are seeking support. For individuals living with gMG and SDOH barriers, targeted PSP approaches may alleviate common challenges associated with gMG, such as receiving a correct diagnosis, finding a specialized care team, accessing treatment (41, 42), and other unique unmet needs (43). Although identifying the needs of historically marginalized populations should be a priority in rare diseases, evidence is still scarce. Those who may need increased support—including people of color, lower socioeconomic status, those on public insurance, and/or with limited healthcare options due to geography—are still critically underrepresented in a majority of studies, in part due to difficulties in communicating and engaging this subpopulation in research initiatives using conventional recruiting approaches (44).

To address these questions, we conducted a sequential mixed-methods study aimed to better characterize how SDOH barriers impact individuals from diverse racial, ethnic, educational, and socioeconomic backgrounds living with gMG across 4 phases of the diagnosis and treatment experience: (1) diagnosis, (2) accessing treatment, (3) initiating treatment, and (4) continuing treatment. Additionally, we investigated their awareness and utilization levels of existing resources, with the aim of identifying and prioritizing interventions that can help mitigate specific challenges associated with SDOH barriers.

## 2. Materials and methods

### 2.1. Study design and ethics

This mixed-methods study included qualitative interviews followed by a cross-sectional multimodal quantitative survey. All participants received compensation for their participation in this study. Records of included participants were held strictly confidential using standard protected health information (PHI) security guidelines under the Health Insurance Portability and Accountability Act of 1996 (HIPAA). Protocols and materials used in the study received Institutional Review Board Approval (IRB#20220823).

### 2.2. Qualitative interviews

#### 2.2.1. Participant recruitment

To capture a diverse cohort of individuals living with gMG who are underrepresented in clinical and interventional studies, a comprehensive list of SDOH factors was identified using resources available from Healthy People 2030 (45) and the World Health Organization (26). Based on these predefined SDOH factors, quotas were implemented to include a balanced spread of various baseline demographic categories including age, gender, employment status, ethnic and racial background, living environment, education status, and annual family income (Supplementary Tables S1, S2). For caregivers and PAG representatives, separate inclusion criteria were also defined (Supplementary Table S3).

Individuals living with gMG, caregivers of those living with gMG, and representatives from gMG PAGs were recruited. The identity of the study sponsor was masked. To overcome the inherent challenges of recruiting a heterogeneous cohort of individuals experiencing SDOH barriers, recruiting strategies included partnering with multiple third-party vendors with robust rare disease networks and collaborating with several gMG PAGs to broaden the reach.

#### 2.2.2. Interviews

Participants who met the inclusion criteria and provided written informed consent were invited to a double-blinded 45-min in-depth qualitative web-assisted phone interview. The moderator was an academic researcher identifying as a woman of color, with expertise engaging and facilitating conversations around SDOH and intersectionality. No identifying information was collected during the interviews.

### 2.3. Survey design

De-identified interview recordings and transcripts were used to categorize, analyze, and curate descriptive themes. Additional information was extracted from a previously described screener (46), existing literature, and a landscape assessment of currently available gMG support resources. These insights were combined to develop questions and relevant response options to identify common concerns and support needs of individuals living with gMG and SDOH barriers (1) overall and (2) at 4 distinct phases throughout the diagnosis and treatment experience defined by: diagnosis, accessing treatment, initiating treatment, and continuing treatment.

### 2.4. Quantitative survey and analysis

#### 2.4.1. Respondent recruitment

Recruitment of individuals living with gMG occurred for a total of 8 weeks, with 2 independent third-party vendors recruiting individuals through their rare disease patient panels and outreach networks, with additional support from US-based gMG PAGs (section 2.2.1). Potential respondents were directed to a screener questionnaire (Supplementary Table S1). Similar to recruitment for the interviews, quotas were predefined to include a balanced spread of various baseline demographic categories including age, gender, employment status, ethnic, and racial background, living environment, education status, and annual family income to ensure a diverse sample of respondents (Supplementary Table S2).

#### 2.4.2. Data collection

Those who fulfilled the inclusion criteria (Supplementary Table S2) were redirected to the online survey, designed to be completed in approximately 20 min. The survey was offered in English or Spanish. As not all individuals living with gMG may be physically and/or emotionally comfortable or capable of completing the survey independently, optional telephone assistance in English or Spanish was offered.

#### 2.4.3. Data analysis

De-identified data were aggregated and analyzed. Exploratory subgroup analyses were conducted using pre-defined SDOH definitions. Results were exported to generate corresponding tables or charts. Quality assurance of data was conducted during fielding, pre-analysis, and post-analysis.

## 3. Results

### 3.1. Qualitative interviews

#### 3.1.1. Participant demographics and characteristics

A total of 15 individuals from across the United States participated in the qualitative interviews: 11 were individuals living with gMG, 2 were caregivers of individuals living with gMG, and 2 were representatives from gMG PAGs. The 11 individuals living with gMG were a diverse group with varying age, racial/ethnic background, gender, living environment, education level, insurance type, and employment status (Supplementary Table S4). Most interviewees identified as non-White/Caucasian (*n* = 8), were covered by public insurance (*n* = 8), and were currently unemployed (*n* = 9) (Supplementary Table S4).

#### 3.1.2. Interview insights

Overall, financial constraints and profound lifestyle shifts were most commonly expressed by interviewees as challenges throughout their experience of receiving a diagnosis and treatment for gMG. Additional barriers such as navigating insurance coverage, maintaining employment, and accessing resources were also commonly mentioned. Interviewees expressed that the logistical and administrative burden of addressing these challenges exacerbated their clinical manifestations of gMG, such as fatigue, especially when combined with treatment side effects and comorbidities. Another critical theme centered around detrimental effects on mental health, including anxiety and fear of exacerbations, social isolation, long periods of emotional distress or despair, and/or suicidal ideation. This mental health burden shifted over time from diagnosis (e.g., confronting new life changes) to post-treatment (e.g., searching for a sense of community and normalcy).

Interviewees discussed that the quality of educational resources they were aware of or were currently using to help understand their diagnosis and treatment options was poor; as a result, feeling misunderstood or burdening others often led to communication challenges. When interviewees were presented with several existing gMG support programs and offerings, overall awareness of these resources was low. Individuals living with gMG, and their caregivers, often sought gMG information and support through social media–based online community groups, with low awareness of other available support such as PAGs.

### 3.2. Quantitative survey

#### 3.2.1. Respondent demographics and characteristics

Thirty-eight individuals living with gMG met the inclusion criteria and completed the web-based survey. Among the 38 respondents, age, racial/ethnic background, gender, living environment, education level, insurance type, and employment status were diverse (Table 1). Notably, 60.5% (*n* = 23) of respondents identified as non-White/Caucasian, and at least 1 participant in every pre-specified racial/ethnic background subgroup completed the survey. Eleven respondents (28.9%) reported high school or General Education Development (GED) as their highest education status, 13 respondents (34.2%) were enrolled in Medicaid, 17 respondents (44.7%) were enrolled in Medicare, and 23 respondents (60.5%) were unemployed. Despite efforts to actively recruit male respondents, significantly more respondents identifying as women were represented (28 women vs. 9 men), with one participant identifying as binary gender nonconforming (Table 1).

**Table 1:** Baseline demographics and characteristics of individuals living with gMG.

		*N* = 38
Age	18–40 years	9
	41–60 years	20
	61–75 years	9
Gender	Men	9
	Women	28
	Binary gender nonconforming	1
Racial/ethnic background	White/Caucasian	15
	Hispanic/Latin@	5
	Black/African American	10
	Native American/Indigenous Person	4
	Asian/Pacific Islander	1
	Middle Eastern or North African	3
Living environment	Urban	13
	Suburban	13
	Rural or small town	12
Education status	High school/GED	11
	Post-secondary education	23
	Prefer not to answer	4
Current insurance type*	Private	7
	Medicaid	13
	Medicare	17
	Other	1
Employment status†	Employed	10
	Unemployed	23
	Retired	5

GED, General Education Development; gMG, generalized myasthenia gravis. *“Private” insurance included commercial and employer-provided insurance. “Other” insurance included Veterans’ Affairs and self-purchased insurance. Individuals who responded with “Other” insurance were excluded from the insurance type-based subgroup analyses. †“Employed” included self-employed. “Unemployed” and “Retired” were combined into “Unemployed” for subsequent subgroup analyses.

#### 3.2.2. Overall concerns, resource usage, and support needs

Overall, financial concerns were highlighted as the most common challenges experienced in daily life, as 76% of respondents reported problems making ends meet at the end of the month, and 50% reported worry or concern that they may not have stable housing (Table 2). While the majority of respondents reported high levels of agency (feeling of control) in healthcare decision-making with their doctors (6 or 7 on a scale of 1 to 7) (Supplementary Figure S1), a distinct cohort of up to 34% of respondents expressed low to neutral (1 to 5 on a scale of 1 to 7) confidence and comfort across facets of their relationship with their doctors, most notably including feeling empowered in managing their gMG (34%) and trust in their doctor’s gMG expertise (34%) (Table 3). Further, 50% of respondents reported low to neutral confidence in navigating their healthcare and treatment experience with ease, and 39% of respondents expressed unmet needs around social support and disease education.

**Table 2:** Overall day-to-day concerns living with gMG.

	*N* = 38
You have had problems **making ends meet** at the end of the month.	76%
You have felt worried/concerned that you may not have **stable housing** that you own, rent, or stay in as a part of a household.	50%
You have felt like you received unequal treatment due to your **socioeconomic status** (eg, level of income).	32%
You have had to skip buying medications or going to doctors’ appointments and/or treatments to **save money**.	32%
You have put off or neglected going to the doctor because of distance or transportation.	32%
The electric, gas, oil, or water company has threatened to shut off services to your home.	29%
You have felt judged due to not understanding something new or unfamiliar to you by someone with more education.	24%
You have felt worried that the place you are living now is making you sick or unsafe (has mold, bugs/rodents, water leaks, not enough heat, lead paint/pipes, lack of smoke detectors).	21%
You have felt like you received unequal treatment due to your race/ethnicity/skin color.	13%
You have had difficulty communicating with someone due to English not being your fist language.	0%

gMG, generalized myasthenia gravis. Respondents were asked to select all statements that were relevant in their day-to-day lives over the past 6 months. Statements are ordered from largest to smallest proportion of respondents reporting the statement as a day-to-day concern. Statements were shown in a randomized order during the questionnaire.

**Table 3:** Overall patient-doctor relationship dynamics, healthcare agency and literacy, sources of gMG information, and perceived quality of gMG information.

	*N* = 38
Low or neutral in patient-doctor relationship *	
My doctor and care team empower me to manage my gMG	34%
I fully trust my doctor’s gMG expertise	34%
I can play an active role in my gMG treatment experience with my doctor	32%
My doctor takes my symptoms seriously when I describe them	29%
I fully trust my doctor’s medical judgement	29%
Low or neutral in healthcare agency and literacy *	
I can navigate my healthcare and treatment experience with ease	50%
I have support from my family, friends, and community to manage my health	39%
I have enough information to manage my health	39%
I have the ability to find reliable health information	39%
I can compare different sources of health information to decide what is best for me	37%
**Most commonly used sources of gMG information†**	
Doctors/healthcare providers/therapists	82%
Internet websites	68%
Social media communities	68%
**Perceived quality of gMG information‡**	
Conflicting (vs. consistent)	29%
Outdated (vs. updated)	16%
Confusing (vs. clear)	16%

gMG, generalized myasthenia gravis. *Percentage shown denotes the proportion of respondents who reported “Low” to “Neutral” agreement with the statement (1 to 5 on a scale of 1 to 7). The 5 statements for which this proportion was highest are shown in this table; full dataset is provided in the Supplementary Material. †The 3 most commonly used sources are shown; full dataset is provided in the Supplementary Material. ‡Percentages shown denotes the proportion of respondents who chose 1 or 2 on a scale of 1 to 7 of agreement with the statement. The 3 statements for which this proportion was highest are shown in this table; full dataset is provided in the Supplementary Figure S1.

Most respondents reported to seek gMG information through their HCPs (82%), followed closely by internet websites and social media–based communities (both 68%) (Table 3). Regarding the quality of currently available gMG information, most respondents had neutral impressions (Supplementary Figure S1). Consistency of information had the most negative perceptions, with 29% of respondents expressing that currently available information from different sources was conflicting rather than consistent (Table 3).

#### 3.2.3. Concerns and support needs by phase of the diagnosis and treatment experience

##### 3.2.3.1. Diagnosis

The most common concerns at the time of diagnosis were how the diagnosis may change their lifestyle (61%) and managing fear and confusion due to not understanding gMG (45%), which highlighted feelings of uncertainty and potential negative effects on mental health (Table 4). Approximately one-third of respondents also expressed logistical challenges, including finding the right specialist (34%) and extended time taken to receive a correct diagnosis (29%) (Supplementary Figure S2A).

**Table 4:** Concerns by phase (diagnosis, accessing treatment, initiating treatment, and continuing treatment).

	*N* = 38
**Diagnosis**	
How your gMG diagnosis might change your lifestyle	61%
Fear and confusion due to not understanding gMG and its symptoms	45%
Managing your gMG diagnosis and care planning due to reduced energy/fatigue	39%
**Accessing treatment**	
Determining if you are qualified for your treatment and how you will pay for it	47%
Managing the logistics of applying for disability and other assistance programs	45%
Mental stress due to uncertainty of how treatment would be paid for	39%
**Initiating treatment**	
Preparing for potential side effects from treatment	71%
Acceptance of the many life changes resulting from gMG diagnosis	66%
Managing your current lifestyle while on treatment	55%
**Continuing treatment**	
Consistent fear of experiencing an unexpected crisis	50%
Quality of life/lifestyle adjustments required to live with gMG for the long term	45%
Challenging maintaining lifestyle given reduced energy levels/fatigue	34%

gMG, generalized myasthenia gravis. Respondents were asked to select 3 statements that were most relevant in each phase. The 3 most commonly reported concerns for each phase are shown in this table; the full dataset is available in the Supplementary Figure S2. Statements were shown in a randomized order during the questionnaire.

Most respondents were unaware of many of the support offerings and resources available through PSPs at diagnosis, aside from general disease information (Table 5 and Supplementary Figure S3A). Reflecting concerns around disease education and mental health, respondents commonly reported that the most valuable resources at this phase would have been nurse navigator support (42%); general disease information resources (42%); information around disease symptoms, testing, and treatment options (37%); and customized mental health services (32%) (Table 6 and Supplementary Figure S4A). Most respondents (71%) preferred to receive these support resources from their doctor, followed by patient support groups (45%) and other individuals living with gMG (34%) (Supplementary Figure S5A).

**Table 5:** Current resource usage by phase (diagnosis, accessing treatment, initiating treatment, and continuing treatment).

	*N* = 38
**Diagnosis**	
*Most commonly used*	
Resources that provide general disease information about gMG	61% used
Resources on commonly used gMG terms to help understand gMG	55% used
*Most unaware*	
Resources that explain diagnostic testing options and how to get tested	63% unaware
Periodic updates sent with gMG information, developments, and resources	63% unaware
**Accessing treatment**	
*Most commonly used*	
Services to verify insurance benefits and coverage	37% used
Financial support from a foundation or advocacy group	32% used
*Most unaware*	
Free medication given while waiting to hear if insurance will cover costs	84% unaware
Copay card to help cover out-of-pocket costs	74% unaware
**Initiating treatment**	
*Most commonly used*	
Packets or brochures with gMG treatment information	63% used
Periodic updates sent with gMG information, developments, and resources	47% used
*Most unaware*	
Nurse case manager to support the beginning of treatment journey	79% unaware
Discussion guides to help discuss treatment options with doctor	61% unaware
**Continuing treatment**	
*Most commonly used*	
Forum where stories of people living with gMG can be shared with others	76% used
Forum where stories of people living with gMG on treatment can be shared	68% used
*Most unaware*	
Resource guide on the steps to take to find a qualified gMG neurologist	66% unaware
Tool to search for nearby infusion centers for treatment using your zip code	58% unaware

gMG, generalized myasthenia gravis. Respondents were asked to select 1 of 3 choices for each of the provided statements describing currently available resources: “Resources used,” “Resources aware of but not used,” and “Resources unaware of.” Statements shown here are the 2 most commonly used and 2 most commonly expressed as unaware of and are paraphrased from actual statements; the full dataset is available in the Supplementary Figure S4. Statements were shown in a randomized order during the questionnaire.

**Table 6:** Most valuable resources by phase (diagnosis, accessing treatment, initiating treatment, and continuing treatment).

	*N* = 38
**Diagnosis**	
Nurse navigator to provide support for newly diagnosed individuals, including referring to resources for mental health	42%
Resources that provide general disease information about gMG	42%
Resources developed by clinical gMG experts that explain gMG symptoms, testing, and treatment options (including benefits and side effects)	37%
**Accessing treatment**	
List of funding sources and assistance programs, with guidance on how to obtain support	39%
Free medication given while waiting to hear if insurance will cover costs	34%
Nurse case manager who can help navigate the insurance process (including working directly with doctors and insurance companies) and help understand potential financial assistance programs	34%
**Initiating treatment**	
Educational resources developed by top clinical gMG experts, explaining gMG symptoms, testing, and treatment options (including benefits and side effects)	42%
Nurse case manager available to support individuals living with gMG starting their treatment journey	39%
Guide on how to manage gMG and additional health concerns (eg, comorbidities)	34%
**Continuing treatment**	
Quick emergency “takeaway” resources for individuals diagnosed with gMG to carry/bring during an exacerbation or crisis	45%
Collection of resources providing tips for practicing better physical and mental health in daily life with gMG	42%
Tools to track symptoms/nurse case manager/social community	34%

gMG, generalized myasthenia gravis. Respondents were asked to select 3 statements that would have been the most valuable in each phase, regardless of whether they were available or utilized. The 3 most commonly reported valuable resources for each phase are shown in this table; statements have been paraphrased here; the full dataset is available in the Supplementary Figure S5. Statements were shown in a randomized order during the questionnaire.

##### 3.2.3.2. Accessing treatment

When seeking access to treatment, the most common concerns were financial, including qualifying and paying for treatment (47%) and navigating the logistics of assistance programs (45%) (Table 4 and Supplementary Figure S2B). Anxiety related to financial concerns was common in this phase (39%).

Despite high levels of financial and access-related concerns, awareness and utilization of available financial resources such as copay cards and free medication was extremely low (Table 5 and Supplementary Figure S3B). Respondents most commonly expressed that a list of funding assistance with guidance on how to obtain support (39%) and free medication (34%) would have been valuable (Table 6 and Supplementary Figure S4B). In addition, nurse case managers trained in various areas such as navigating insurance (34%), identifying resources for Medicaid or Medicare (26%), and/or connecting individuals to mental health services and community resources (24%) were also expressed as resources that would have been valuable. Most respondents preferred to obtain these resources from their doctor (63%) or patient support groups (45%), followed by PAGs (32%) and manufacturers (29%) (Supplementary Figure S5B).

##### 3.2.3.3. Initiating treatment

Preparing for potential side effects (71%), acceptance of lifestyle changes (66%), and managing their current lifestyle while on treatment (55%) comprised the most common concerns at treatment initiation, highlighting the sensitive nature of initiating treatment and its potential effects on anxiety (Table 4 and Supplementary Figure S2C).

The majority of respondents had greater awareness and utilization of available treatment- and lifestyle-related resources when initiating treatment; however, awareness was low around nurse case managers and treatment option discussion guides (Table 5 and Supplementary Figure S3C). Resources that would have been the most valuable during this phase were most commonly reported as educational resources (42%), nurse case managers (39%), and a guide to managing comorbidities (34%) (Table 6 and Supplementary Figure S4C). Most respondents preferred to obtain these resources from their doctor (71%) or patient support groups (42%), followed by PAGs (29%) (Supplementary Figure S5C).

##### 3.2.3.4. Continuing treatment

When continuing treatment, common concerns included consistent fear of experiencing an unexpected crisis (50%) and considering long-term lifestyle adjustments (45%), signifying that treatment-related challenges and anxieties continue even after the treatment initiation phase (Table 4 and Supplementary Figure S2D).

Most respondents engaged with patient communities in this phase for support, with lower awareness of other available tools such as guides for finding a qualified gMG neurologist and search tools to find the closest infusion centers (Table 5 and Supplementary Figure S3D). Consistent with common concerns in this phase, resources that would have been the most valuable were quick emergency resources to bring during a crisis or exacerbation (45%) and resources providing tips for maintaining physical and mental health in gMG (42%) (Table 6 and Supplementary Figure S4D). Respondents preferred to receive these resources from their doctors (66%) or patient support groups (50%), followed by other individuals living with gMG (32%) and PAGs (26%) (Supplementary Figure S5D).

## 4. Discussion

In this study, we aimed to better characterize the experiences of individuals living with gMG who belong to historically underrepresented communities based on various SDOH challenges. We utilized a unique approach to capture their awareness and utilization of currently available PSPs, with the aim to better tailor PSPs to their support needs. This study begins to address a critical knowledge gap, highlighting previously underexplored perspectives to understand how support for diagnosed individuals, their social environment, and the healthcare system can be further improved for rare diseases such as gMG.

### 4.1. Key learning 1: Individuals living with gMG and SDOH barriers face a complex set of challenges across diagnosis, accessing treatment, initiating treatment, and continuing treatment; most of these challenges centered around financial concerns and mental health

Among the diverse study cohort, financial concerns and mental health were expressed as the most common gMG-related concerns. As expected, financial concerns were emphasized while accessing treatment. Negative effects on mental health were common throughout all phases but associated with different underlying concerns at each phase, demonstrating the multifaceted and dynamic nature of living with gMG. These core concerns evolved from fear and confusion toward understanding gMG at diagnosis, to financial concerns when accessing treatment, to short–(e.g., anticipation of treatment side effects) and long-term (e.g., anticipation of experiencing unexpected crises) lifestyle changes when initiating and continuing treatment. Although these challenges have been reflected in more general populations of individuals living with gMG (15, 41, 47), financial and mental health challenges may be further amplified for those living with SDOH barriers. As depressive symptoms can negatively affect HRQoL in gMG (15, 48–50), mental health should be particularly monitored in underrepresented communities on a consistent basis.

### 4.2. Key learning 2: Well-publicized and easy-to-access educational and financial resources would be most valuable for individuals living with gMG and SDOH barriers

Our results highlighted an overall low awareness and utilization of existing support services among individuals living with gMG and SDOH barriers. This could be attributed to not only the general scarcity of gMG-specific resources and PSPs as in many rare diseases, but also systemic barriers to finding, accessing, and/or receiving existing services for those experiencing SDOH barriers. Although educational resources were commonly perceived as the most valuable resources throughout the diagnosis and treatment continuum, some negative perceptions were captured on the consistency of available information, highlighting the importance of continuing to improve exposure and user-friendliness of accurate and well-curated resources. The high perceived value of general disease information that discusses symptoms, diagnostic testing, and treatment options at diagnosis and treatment initiation illustrates the need to deliver high-quality information during this critical time.

Particularly when accessing treatment, financial support was expressed as most valuable. Despite the high perceived value associated with financial support resources such as funding assistance and free medication, respondents had a low awareness of existing programs that offer such support. To better meet the needs of individuals living with gMG and SDOH barriers, potential systemic barriers to access these resources should be further explored and addressed.

### 4.3. Key learning 3: “High-touch” and tailored support programs may be better suited to improve outcomes for underserved populations

Throughout the diagnosis and treatment experience, respondents preferred to receive support resources from HCPs, closely followed by patient support groups. While the preference for HCPs is not surprising, heavy reliance on patient support groups could reflect unmet needs for diverse disease-related, cultural, and personal support, especially in underserved and underrepresented communities (51).

Notably, nurse case managers—who offer personalized guidance for individuals living with gMG in a variety of realms, including disease education, navigating insurance, tracking symptoms, and sources for financial assistance—were commonly perceived as a valuable resource. However, their current awareness and utilization were low, which could in part be due to the lack of their availability at healthcare facilities where individuals receive their care. PSPs offering nurse case managers and similar services that are high in “human touch” have been shown to increase medication adherence, satisfaction, HRQoL, and/or lower total medical costs in many chronic diseases including diabetes, hypertension, cancer, and autoimmune conditions (39, 40, 52, 53). Across these therapy areas, case management has been associated with decreased emergency care utilization, hospital admissions, readmissions, and length of stay, reducing healthcare system costs (52). In one study examining patient-centered coaching interventions in 321 discharged Medicare users, over $3,700 of cost savings per patient were reported over 6 months compared to controls, with no cost shifting observed to other types of healthcare utilization (53). Moreover, in individuals living with chronic autoimmune diseases, participation in a PSP with personalized 1-to-1 support was associated with 29% higher medication adherence, 22% lower discontinuation rates, and 35% lower disease-related medical costs compared with non-PSP controls (40). Similar PSP benefits can be expected in gMG, as our results highlight unmet needs for additional empowerment and education to allow individuals to be better equipped to manage their diagnosis and disease.

Such resources can be especially valuable in rare diseases as HCPs may not always be well-equipped to identify and address SDOH challenges with limited time and resources available to support individuals’ holistic needs beyond the clinical realm. Improved visibility and access to “high-touch” PSPs in gMG can provide additional support for social workers and community resources who HCPs often rely on to address these needs. For individuals with diabetes, involvement of case managers increased activation of social resources, allowing individuals with lower income and educational levels to achieve the same treatment benefits as those observed in individuals with higher income and educational levels (52). Increased utilization of such “high-touch” support can help individuals living with gMG and experiencing SDOH barriers achieve better outcomes, as self-efficacy and tangible support can be independent predictors of mental health status in gMG (54).

### 4.4. Key learning 4: Deliberately increasing visibility and expanding inclusivity of support networks can further benefit individuals living with gMG and SDOH barriers

With diversity, equity, and inclusion becoming a greater priority in the United States (55), socially conscious innovations are needed to improve representation and accommodation of historically marginalized individuals in health programs that have long been subject to systemic practices that perpetuate health inequities (56). Our research informs 2 priorities to improve support for individuals living with gMG and SDOH barriers.

First, awareness and utilization of existing support services should be increased. To accomplish this, education on available support services should be improved throughout the larger gMG ecosystem, including HCPs, PAGs, social workers, pharmacies, infusion centers, and health plans. This can help provide timely access of support services and resources, such as fact-checked educational materials, trained nurse case managers, and financial assistance, to individuals living with gMG who need them. For HCPs, forming dedicated multidisciplinary care teams or clinics may also enhance gMG knowledge-sharing between clinicians, nurses, rehabilitation specialists, physical/occupational therapists, dieticians, speech-language pathologists, and other professionals so that individuals can be connected with available resources as soon as they are diagnosed with gMG.

Secondly, updated, high-quality educational resources offered by support programs such as PSPs should be disseminated via an increasingly multichannel approach to accommodate a diverse range of communication preferences. These can include treatment information telephone hotlines, digital symptom trackers, educational webinars, online nurse case managers, and social media campaigns. Importantly, updated offline materials such as pamphlets and printed guides should also be offered at healthcare sites and by mail for those who may prefer them or have limited digital literacy. Increased collaborations throughout the gMG ecosystem can be leveraged to share these resources robustly across communities of individuals living with gMG.

To continue better addressing the needs of individuals living with gMG and SDOH barriers in the longer term, financial investments to support initiatives that can improve access to treatment for vulnerable populations living with gMG should be prioritized. These can include increasing availability of multilingual assistance, localized educational programs, and public and private funding assistance. In addition, future research should better identify individuals who may need additional and/or specific types of assistance related to SDOH on a larger scale with the use of large databases such as electronic health records. To gather more data, enrollment of historically underrepresented populations should be increased in gMG research. More broadly, the importance of early detection, early diagnosis, and better screening of mental health symptoms in gMG should be further studied.

### 4.5. Limitations

This cross-sectional study was designed to characterize experiences of a specific cohort, and these data are not intended to be comparative with that of a general population of individuals living with gMG. Expanded longitudinal observational studies are needed to better understand its implications, to further contextualize how SDOH barriers may contribute to health inequities in gMG, and how these data may also be relevant to broader rare diseases. Although SDOH-specific differences are of high relevance, subgroup analyses were exploratory. The sample size was not powered for statistical testing and considerable variability of responses among the sample led to the absence of any strong patterns discerned between SDOH subgroups. The heterogeneity of responses could be in part attributed to the variable (between individuals) and fluctuating (within an individual) nature of gMG, as well as the complex intersectionality of SDOH factors for each individual among the limited study sample. Finally, we used primarily web-based data collection for both recruitment and study phases. Although measures were taken to minimize selection bias for digital literacy through offering telephone assistance to complete the survey (in English and Spanish), the study cohort may have been enriched with individuals with access to basic web/phone-based services at the minimum.

## 5. Conclusion

Our results highlight distinct concerns and unmet needs among a diverse population of individuals with gMG living with various SDOH barriers. Financial and mental health concerns were common overall; additionally, barriers to access and poor disease education were also common, depending on the phase of the diagnosis and treatment experience. Although resource and support needs reflected their concerns, awareness and utilization of existing PSPs were low, emphasizing the need for further targeted, specific, accessible, and well-publicized support. To improve the experiences of historically marginalized individuals in the healthcare system, the larger gMG support network should continue to spotlight these communities to work together to provide further evolved customized and localized support focused on the specific needs of these communities.

## Data availability statement

The raw data supporting the conclusions of this article will be made available by the authors, without undue reservation.

## Ethics statement

The studies involving human participants were reviewed and approved by WCG IRB, Puyallup, WA, United States. The patients/participants provided their written informed consent to participate in this study.

## Author contributions

TH, KP, CB, SS, CZ, DM, AW, and GP were involved in developing the concept and designing the study methodology. SS, CZ, DM, and AW oversaw data collection and analysis, and all authors were involved in interpretation. MS drafted the manuscript. All authors contributed to the article and approved the submitted version.

## Funding

This study was funded by argenx US Inc. (Boston, MA, United States). The funder was involved in the study design, interpretation of aggregate results, manuscript reviews, and the decision to submit for publication. The funder did not have access to raw data.

## Conflict of interest

TH, KP, CB, and GP are employed by argenx (Boston, MA, United States). AA is a Patient Education Speaker for argenx. AH receives research support from Alexion/AstraZeneca, argenx, UCB, Immunovant, Regeneron, Cabaletta Bio, Viela Bio/Horizon, and Genentech, and has received honoraria from UCB, argenx, Alexion, Immunovant, Regeneron, and Genentech/Roche. SS, CZ, DM, MS, and AW are employed by ZS Associates (Evanston, IL, United States) and serve as paid consultants for argenx.

## Publisher’s note

All claims expressed in this article are solely those of the authors and do not necessarily represent those of their affiliated organizations, or those of the publisher, the editors and the reviewers. Any product that may be evaluated in this article, or claim that may be made by its manufacturer, is not guaranteed or endorsed by the publisher.

## Abbreviations

GED: General Education Development
gMG: generalized MG; HCP, healthcare provider
HIPAA: Health Insurance Portability and Accountability Act of 1996
MG: myasthenia gravis
PAG: patient advocacy group
PHI: protected health information
PSP: patient support program
SDOH: social determinants of health.
