# Supplementary material for: Impact of social determinants of health on individuals living with generalized myasthenia gravis and implications for patient support programs
Source: Front Public Health. 2023 May 19;11:1147489. doi: 10.3389/fpubh.2023.1147489 (PMC10235801; doi:10.3389/fpubh.2023.1147489)
Supplement: Supplementary file 1 [file Data_Sheet_1.docx]

Supplementary Material

**Supplementary tables and figures included in this file:**

Table S1: Screener questions used in recruitment of individuals living with gMG.

Table S2: Inclusion criteria and custom screening parameters for patients living with gMG (Uniform for both interviews and survey unless otherwise noted)

Table S3: Inclusion criteria for caregivers and PAG representatives

Table S4: Baseline demographics and characteristics of interview participants

Figure S1: Overall patient-doctor relationship dynamics, healthcare agency and literacy, sources of gMG information, and perceived quality of gMG information

Figure S2: Concerns by phase, subgroup analysis

Figure S3: Current resource usage by phase (diagnosis, accessing treatment, initiating treatment, and continuing treatment)

Figure S4: Most valuable resources by phase, subgroup analysis

Figure S5: Preferred source of information and support by phase (diagnosis, accessing treatment, initiating treatment, and continuing treatment)

# Table S1: Screener questions used in recruitment of individuals living with gMG.

| **Question** | **Response choices** |
| --- | --- |
| What is your current age? | -Fill in response ##: _________  - Prefer not to answer |
| Which best reflects your current gender? Select one | -Male  -Female  -Gender binary non confirming  -Other(Please Specify: ____)  -Prefer not to say |
| Which of the following best describes your race or ethnicity? Please select all that apply. | -White or Caucasian  -Hispanic or Latino/a  -African American or Black  -Asian or Pacific Islander  -Native American  -Middle Eastern or North African  -Other  -Prefer not to answer |
| Which of the following best describes where you currently live? | -In a big city  -In a small town  -In a suburb  -In a rural area |
| What is the highest level of education you have completed? Please select one. | -Some high school  -High school  -GED  -Some college  -Associates degree  -Bachelor’s degree  -Advanced degree (MD, MBA, JD, etc.)  -Prefer not to answer |
| What type of healthcare insurance do you have? Please select all that apply. | -Insurance coverage through a current/former employer  -Insurance coverage through a spouse’s employer  -Medicare  -Medicaid  -Self-purchased individual or family insurance plan  -Veteran’s Administration (VA) / CHAMPUS -Other (Please Specify: ____)  -None |
| What is your current employment status? Please select one. | -Employed full time  -Employed part time  -Self-employed  -Not employed  -Student  -Retired  -Prefer not to answer |

Table S1 shows representative screener questions relevant to the study. The full screener is available from the corresponding author upon reasonable request.

# Table S2: Inclusion criteria and custom screening parameters for patients living with gMG (Uniform for both interviews and survey unless otherwise noted)

| **Inclusion criteria for patients living with gMG** | **Custom screening parameters to ensure diverse sample** |
| --- | --- |
| Currently living with gMG | All |
| Have been or currently on treatment to manage gMG | All |
| Currently reside in US state or territory | All |
| Aged 18-75 years | Screened to include a distribution of patients aged 18-39, 40-59, and 60-75 years of age |
| Able to disclose gender | Screened to include a balanced distribution of men and women |
| Able to disclose employment status | Screened to include a distribution of employed and unemployed individuals |
| Able to disclose ethnic and racial background | Screened to include a distribution of patients identifying as White or Caucasian, Hispanic or Latin@, African American or Black, Native American or Indigenous Person, Asian or Pacific Islander, and Middle Eastern or North African |
| Able to disclose living environment | Screened to include a distribution of patients identifying as living in an urban, suburban, or rural environment |
| Able to disclose education status | **Interviews:** Screened to include a distribution of patients with last known education status of high school/GED, Associate degree/vocational/some college, or 4-year college/graduate degree  **Survey:** Screened to include a distribution of patients with last known education status of no postsecondary degree, some college, or Associate degree |
| Able to disclose income, and annual family income is ≤$100,000 | For participants identifying as White or Caucasian, annual family income was capped at ≤$75,000 for inclusion in the study |

GED, General Education Development; gMG, generalized myasthenia gravis; US, United States.

# Table S3: Inclusion criteria for caregivers and PAG representatives

| **Inclusion criteria for caregivers of patients living with gMG (n=2)** | **Inclusion criteria for gMG PAG representatives (n=2)** |
| --- | --- |
| - Currently caring for someone living with gMG aged 18-75 years - Patient under care has used at least 1 treatment for gMG - Is a family, relative, friend, or professional caregiver of a patient living with gMG - Gives regular care or ad hoc support for a patient with gMG - Last known education status of patient being cared for is high school, GED, some college, or Associate’s degree - Annual household income of patient being cared for is ≤$100,000 (or ≤$75,000 if patient is While or Caucasian) - Able to disclose gender and employment status of the patient cared for | - Resides in a US state or territory - Had served or is serving as a member of a diverse community of patients with gMG, including members of any or all diverse racial and ethnic backgrounds - Has been working with or for a gMG PAG for ≥3 years - Personally interacted with ≥5 patients receiving assistance or services for gMG in the last 60 days |

gMG, generalized myasthenia gravis; PAG, patient advocacy group.

# Table S4: Baseline demographics and characteristics of interview participants

|  |  | Interviews*, n (N=11) |
| --- | --- | --- |
| Age | 18-40 years | 3 |
|  | 41-60 years | 7 |
|  | 61-75 years | 1 |
| Gender | Men | 4 |
|  | Women | 7 |
|  | Binary gender nonconforming | 0 |
| Racial/ethnic background | White/Caucasian | 3 |
|  | Hispanic/Latin@ | 2 |
|  | Black/African American | 4 |
|  | Native American/Indigenous Person | 0 |
|  | Asian/Pacific Islander | 2 |
|  | Middle Eastern or North African | 0 |
| Living environment | Urban | 4 |
|  | Suburban | 3 |
|  | Rural or small town | 4 |
| Education status | High school/GED (General Education Development) | 2 |
|  | Postsecondary education | 9 |
|  | Prefer not to answer | 0 |
| Current insurance type† | Private | 1 |
|  | Medicaid | 5 |
|  | Medicare | 3 |
|  | Other | 3 |
| Employment status‡ | Employed | 2 |
|  | Unemployed | 9 |
|  | Retired | 0 |

*Baseline demographics and characteristics are only shown for those who were individuals living with gMG (n=11), and not shown for caregivers (n=2) and PAG representatives (n=2). †Insurance type totals may not add up to the total sample size as respondents could choose multiple options if applicable. ”Private” insurance included commercial and employer-provided insurance. “Other” insurance included Veterans’ Affairs and self-purchased insurance. Individuals who responded with “Other” insurance were excluded from the insurance type–based subgroup analyses. ‡”Employed” included self-employed. “Unemployed” and “Retired” were combined into “Unemployed” for subsequent subgroup analyses.

# Figure S1: Overall patient-doctor relationship dynamics, healthcare agency and literacy, sources of gMG information, and perceived quality of gMG information

A. Patient-doctor relationship B. Healthcare agency and literacy

C. Sources of gMG information D. Perceived quality of gMG information

# Figure S2: Concerns by phase, subgroup analysis

1. Diagnosis

|  | Overall | Area of Residence | | Insurance Status | | | Education Level | | Ethnicity | | Employment Status | |
| --- | --- | --- | --- | --- | --- | --- | --- | --- | --- | --- | --- | --- |
|  |  | Rural or Small town | Urban or Suburban | Medicare | Medicaid | Private | Post-secondary | High school or GED | White/Caucasian | Non-White/Caucasian | Employed | Not Employed |
| How your MG diagnosis might change your lifestyle | 61% | 58% | 62% | 35% | 77% | 86% | 65% | 55% | 53% | 65% | 70% | 57% |
| Fear & confusion due to not understanding MG and its symptoms | 45% | 33% | 50% | 41% | 54% | 43% | 57% | 36% | 47% | 43% | 60% | 39% |
| Managing your MG diagnosis and care planning due to reduced energy levels / fatigue | 39% | 42% | 38% | 41% | 46% | 14% | 30% | 55% | 40% | 39% | 30% | 43% |
| Finding the right specialist to help manage your MG | 34% | 50% | 27% | 41% | 23% | 43% | 35% | 27% | 40% | 30% | 50% | 29% |
| How your MG diagnosis might impact finances | 34% | 25% | 38% | 41% | 23% | 29% | 30% | 45% | 40% | 30% | 10% | 43% |
| The time it took to receive a correct diagnosis | 29% | 17% | 35% | 29% | 23% | 43% | 35% | 18% | 13% | 39% | 50% | 21% |
| The ability to make medical decisions with limited knowledge about MG without support | 16% | 25% | 12% | 12% | 23% | 14% | 13% | 18% | 20% | 13% | 20% | 14% |
| Explaining your MG diagnosis to your other doctors who are managing other non-MG related conditions | 16% | 17% | 15% | 18% | 23% | 0% | 17% | 9% | 20% | 13% | 0% | 21% |
| Being able to communicate symptoms to your doctor | 11% | 17% | 8% | 18% | 8% | 0% | 0% | 36% | 13% | 9% | 0% | 14% |
| Other | 8% | 8% | 8% | 18% | 0% | 0% | 4% | 0% | 7% | 9% | 10% | 7% |
| Low or a lack of empathy from your doctor | 5% | 0% | 8% | 0% | 0% | 29% | 9% | 0% | 0% | 9% | 0% | 7% |
| Connecting to others living with MG/MG-related patient support groups | 3% | 8% | 0% | 6% | 0% | 0% | 4% | 0% | 7% | 0% | 0% | 4% |
| n | 38 | 12 | 26 | 17 | 13 | 7 | 23 | 11 | 15 | 23 | 10 | 28 |

1. Accessing treatment

|  | Overall | Area of Residence | | Insurance Status | | | Education Level | | Ethnicity | | Employment Status | |
| --- | --- | --- | --- | --- | --- | --- | --- | --- | --- | --- | --- | --- |
|  |  | Rural or Small town | Urban or Suburban | Medicare | Medicaid | Private | Post-secondary | High school or GED | White/Caucasian | Non-White/Caucasian | Employed | Not Employed |
| Determining if you are qualified for your treatment and how will you pay for it | 47% | 33% | 54% | 41% | 46% | 57% | 43% | 45% | 40% | 52% | 60% | 43% |
| Managing the logistics of applying for disability and other assistance programs | 45% | 50% | 42% | 47% | 62% | 14% | 52% | 27% | 53% | 39% | 40% | 46% |
| Mental stress due to uncertainty of how treatment would be paid for | 39% | 33% | 42% | 29% | 46% | 57% | 52% | 27% | 40% | 39% | 50% | 36% |
| Managing the logistics of receiving financial authorization and approval for your treatment by your insurance | 34% | 42% | 31% | 41% | 31% | 14% | 22% | 55% | 47% | 26% | 10% | 43% |
| Limitations to your household income that made the ability to pay for treatment difficult (n=38) | 29% | 33% | 27% | 41% | 23% | 14% | 26% | 36% | 33% | 26% | 30% | 29% |
| Changes in your insurance coverage, due to employment | 21% | 8% | 27% | 18% | 8% | 57% | 30% | 0% | 13% | 26% | 30% | 18% |
| Finding a doctor who accepted your insurance | 16% | 17% | 15% | 18% | 23% | 0% | 17% | 9% | 0% | 26% | 10% | 18% |
| None of the above | 5% | 8% | 4% | 6% | 0% | 14% | 4% | 9% | 7% | 4% | 10% | 4% |
| Other | 0% | 0% | 0% | 0% | 0% | 0% | 0% | 0% | 0% | 0% | 0% | 0% |
| n | 38 | 12 | 26 | 17 | 13 | 7 | 23 | 11 | 15 | 23 | 10 | 28 |

1. Initiating treatment

|  | Overall | Area of Residence | | Insurance Status | | | Education Level | | Ethnicity | | Employment Status | |
| --- | --- | --- | --- | --- | --- | --- | --- | --- | --- | --- | --- | --- |
|  |  | Rural or Small town | Urban or Suburban | Medicare | Medicaid | Private | Post-secondary | High school or GED | White/Caucasian | Non-White/Caucasian | Employed | Not Employed |
| Preparing for potential side effects from treatment | 71% | 83% | 65% | 59% | 77% | 86% | 65% | 73% | 67% | 74% | 80% | 68% |
| Acceptance of the many life changes resulting from your MG diagnosis | 66% | 58% | 69% | 53% | 77% | 86% | 70% | 73% | 60% | 70% | 60% | 68% |
| Managing your current lifestyle while on treatment | 55% | 50% | 58% | 47% | 62% | 57% | 57% | 64% | 60% | 52% | 60% | 54% |
| Feeling burdened to understand MG in-depth with little support | 32% | 25% | 35% | 35% | 31% | 29% | 43% | 9% | 27% | 35% | 30% | 32% |
| Lack of support during this transition from family and friends | 32% | 33% | 31% | 41% | 31% | 14% | 30% | 27% | 33% | 30% | 40% | 29% |
| Paying for treatment to manage my MG | 26% | 33% | 23% | 35% | 8% | 29% | 22% | 36% | 40% | 17% | 20% | 29% |
| Managing comorbidities in addition to MG | 11% | 8% | 12% | 18% | 8% | 0% | 9% | 9% | 13% | 9% | 10% | 11% |
| Difficulty finding a qualified treatment center close to home | 5% | 8% | 4% | 6% | 8% | 0% | 4% | 9% | 0% | 9% | 0% | 7% |
| Other | 3% | 0% | 4% | 6% | 0% | 0% | 0% | 0% | 0% | 4% | 0% | 4% |
| None | 0% | 0% | 0% | 0% | 0% | 0% | 0% | 0% | 0% | 0% | 0% | 0% |
| n | 38 | 12 | 26 | 17 | 13 | 7 | 23 | 11 | 15 | 23 | 10 | 28 |

1. Continuing treatment

|  | Overall | Area of Residence | | Insurance Status | | | Education Level | | Ethnicity | | Employment Status | |
| --- | --- | --- | --- | --- | --- | --- | --- | --- | --- | --- | --- | --- |
|  |  | Rural or Small town | Urban or Suburban | Medicare | Medicaid | Private | Post-secondary | High school or GED | White/Caucasian | Non-White/Caucasian | Employed | Not Employed |
| Consistent fear of experiencing an unexpected crisis | 50% | 42% | 54% | 41% | 62% | 43% | 52% | 45% | 53% | 48% | 50% | 50% |
| Quality of life/ lifestyle adjustments required to live with MG for the long term | 45% | 42% | 46% | 35% | 46% | 57% | 52% | 45% | 47% | 43% | 50% | 43% |
| Challenges maintaining your lifestyle given your reduced energy levels / fatigue | 34% | 25% | 38% | 41% | 31% | 29% | 26% | 45% | 40% | 30% | 40% | 32% |
| Rediscovering your “new normal” routine, sense of self and independence | 32% | 25% | 35% | 12% | 46% | 57% | 30% | 27% | 20% | 39% | 50% | 25% |
| Lack of support from friends and family members who may struggle to understand the consequences of living with MG | 24% | 25% | 23% | 18% | 38% | 14% | 17% | 36% | 13% | 30% | 10% | 29% |
| The fear of causing loved ones and caregivers to burnout or be burdened | 29% | 25% | 31% | 41% | 15% | 29% | 43% | 9% | 40% | 22% | 40% | 25% |
| Loss of coverage for treatment or not being able to afford medication | 21% | 17% | 23% | 29% | 8% | 29% | 22% | 9% | 13% | 26% | 20% | 21% |
| Managing the changes in your personal relationships due to MG | 16% | 42% | 4% | 18% | 8% | 14% | 13% | 27% | 27% | 9% | 10% | 18% |
| Having to be prepared to explain your MG condition to other health professional who are unfamiliar | 13% | 25% | 8% | 29% | 0% | 0% | 9% | 18% | 20% | 9% | 10% | 14% |
| Finding a community for people living with MG | 8% | 8% | 8% | 12% | 8% | 0% | 9% | 0% | 0% | 13% | 0% | 11% |
| Lack of access to ongoing quality healthcare due to distance | 5% | 0% | 8% | 6% | 8% | 0% | 4% | 0% | 0% | 9% | 0% | 7% |
| Finding acceptance in the permanent nature of the MG | 5% | 8% | 4% | 6% | 0% | 14% | 4% | 9% | 7% | 4% | 10% | 4% |
| None of the above | 0% | 0% | 0% | 0% | 0% | 0% | 0% | 0% | 0% | 0% | 0% | 0% |
| Other | 0% | 0% | 0% | 0% | 0% | 0% | 0% | 0% | 0% | 0% | 0% | 0% |
| n | 38 | 12 | 26 | 17 | 13 | 7 | 23 | 11 | 15 | 23 | 10 | 28 |

# Figure S3: Current resource usage by phase (diagnosis, accessing treatment, initiating treatment, and continuing treatment)

A B

C D

Respondents were asked to select 1 of the 3 choices for each statement. Statements were shown in a randomized order.

# Figure S4: Most valuable resources by phase, subgroup analysis

1. Diagnosis

|  | Overall | Area of Residence | | Insurance Status | | | Education Level | | Ethnicity | | Employment Status | |
| --- | --- | --- | --- | --- | --- | --- | --- | --- | --- | --- | --- | --- |
|  |  | Rural or Small town | Urban or Suburban | Medicare | Medicaid | Private | Post-secondary | High school or GED | White/Caucasian | Non-White/Caucasian | Employed | Not Employed |
| Nurse navigator to provide support and answer questions to newly diagnosed patients, including referring to resources for mental health | 42% | 25% | 50% | 41% | 54% | 29% | 43% | 27% | 47% | 39% | 40% | 43% |
| Resource for individuals living with MG that provides general disease information about MG | 42% | 50% | 38% | 41% | 62% | 14% | 39% | 55% | 40% | 35% | 50% | 32% |
| Resource for individuals living with MG, that is developed by clinical MG experts that explains MG symptoms, testing, and treatment options (incl. benefits and side effects) | 37% | 8% | 50% | 29% | 46% | 29% | 35% | 36% | 40% | 43% | 30% | 46% |
| Customized MG-related mental health services | 32% | 42% | 27% | 41% | 15% | 43% | 39% | 27% | 47% | 22% | 30% | 32% |
| Portal to be connected to community organizations that support people living with MG | 29% | 25% | 31% | 12% | 46% | 29% | 30% | 36% | 20% | 35% | 20% | 32% |
| Discussion guide to help you discuss your MG symptoms with your doctor and how these symptoms impact daily life | 24% | 42% | 15% | 24% | 23% | 29% | 22% | 27% | 13% | 30% | 10% | 29% |
| Resource on commonly used MG terms to help individuals living with MG and their loved ones better understand MG | 21% | 25% | 19% | 24% | 23% | 14% | 26% | 18% | 33% | 13% | 30% | 18% |
| Comprehensive list providing information about different advocacy organizations for the MG community that individuals living with MG can connect with | 21% | 25% | 19% | 24% | 8% | 29% | 22% | 18% | 27% | 17% | 20% | 21% |
| Resource to guide conversations with your employer about your MG diagnosis and rights regarding employment | 18% | 8% | 23% | 18% | 8% | 43% | 26% | 0% | 20% | 17% | 50% | 7% |
| Periodic updates sent to you with the latest information, developments and resources that try to talk about the unique ways MG can affect your life | 13% | 8% | 15% | 0% | 15% | 43% | 13% | 9% | 7% | 17% | 20% | 11% |
| Other | 8% | 17% | 4% | 18% | 0% | 0% | 0% | 9% | 7% | 9% | 0% | 11% |
| Resource to connect patients with a doctor using telemedicine | 5% | 0% | 8% | 12% | 0% | 0% | 4% | 9% | 0% | 9% | 0% | 7% |
| n | 38 | 12 | 26 | 17 | 13 | 7 | 23 | 11 | 15 | 23 | 10 | 28 |

1. Accessing treatment

|  | Overall | Area of Residence | | Insurance Status | | | Education Level | | Ethnicity | | Employment Status | |
| --- | --- | --- | --- | --- | --- | --- | --- | --- | --- | --- | --- | --- |
|  |  | Rural or Small town | Urban or Suburban | Medicare | Medicaid | Private | Post-secondary | High school or GED | White/Caucasian | Non-White/Caucasian | Employed | Not Employed |
| List of funding sources and assistance programs, with guidance on how to obtain support | 39% | 50% | 35% | 35% | 31% | 57% | 35% | 27% | 33% | 43% | 50% | 36% |
| Free medication given while waiting to hear if insurance will cover costs | 34% | 25% | 38% | 29% | 62% | 0% | 39% | 36% | 40% | 30% | 20% | 39% |
| ‘Nurse Case Manager’ who can help navigate the insurance process (including working directly with doctors and insurance companies) and help understand potential financial assistance programs | 34% | 25% | 38% | 24% | 38% | 57% | 43% | 9% | 27% | 39% | 50% | 29% |
| A ‘Nurse Case Manager’ trained to identify resources to targeted support for Medicaid/Medicare coverage | 26% | 17% | 31% | 53% | 0% | 14% | 22% | 18% | 27% | 26% | 30% | 25% |
| ‘Nurse Case Managers’ trained to connect patients to mental health services and patient community resources | 24% | 17% | 27% | 24% | 15% | 43% | 39% | 0% | 20% | 26% | 30% | 21% |
| Help/support in the event of treatment denial (eg, due to insurance issues, etc) | 24% | 33% | 19% | 12% | 31% | 29% | 26% | 27% | 27% | 22% | 20% | 25% |
| Financial assistance for individuals without insurance | 21% | 17% | 23% | 6% | 46% | 14% | 17% | 36% | 13% | 26% | 10% | 25% |
| Co-pay card to help cover out-of-pocket costs | 18% | 8% | 23% | 18% | 8% | 43% | 30% | 0% | 20% | 17% | 20% | 18% |
| Services to help with getting insurance approval and troubleshooting issues | 18% | 25% | 15% | 6% | 31% | 14% | 17% | 18% | 20% | 17% | 20% | 18% |
| Services to verify insurance benefits and coverage | 16% | 0% | 23% | 12% | 23% | 14% | 17% | 9% | 20% | 13% | 30% | 11% |
| Guide for managing insurance coverage that is specifically tailored for Medicaid/Medicare coverage | 13% | 17% | 12% | 18% | 15% | 0% | 4% | 27% | 20% | 9% | 10% | 14% |
| Reminders to renew or re-enroll in financial assistance programs | 8% | 17% | 4% | 12% | 0% | 14% | 9% | 9% | 13% | 4% | 10% | 7% |
| n | 38 | 12 | 26 | 17 | 13 | 7 | 0-23 | 0-11 | 15 | 23 | 10 | 28 |

1. Initiating treatment

|  | Overall | Area of Residence | | Insurance Status | | | Education Level | | Ethnicity | | Employment Status | |
| --- | --- | --- | --- | --- | --- | --- | --- | --- | --- | --- | --- | --- |
|  |  | Rural or Small town | Urban or Suburban | Medicare | Medicaid | Private | Post-secondary | High school or GED | White/Caucasian | Non-White/Caucasian | Employed | Not Employed |
| Educational resource for individuals living with MG, developed by top clinical MG experts, explaining MG symptoms, testing, and treatment options (incl. benefits and side effects) | 42% | 42% | 42% | 29% | 46% | 57% | 57% | 27% | 60% | 30% | 50% | 39% |
| ‘Nurse Case Manager’ available to support individual living with MG starting their treatment journey | 39% | 25% | 46% | 53% | 31% | 29% | 48% | 9% | 40% | 39% | 50% | 36% |
| Guide on how to manage MG and additional health concerns (eg, diabetes, asthma, high blood pressure) | 34% | 33% | 35% | 29% | 38% | 29% | 30% | 36% | 20% | 43% | 40% | 32% |
| Flexible options for treatment at infusion centers close to home | 29% | 25% | 31% | 35% | 15% | 43% | 22% | 36% | 13% | 39% | 30% | 29% |
| ‘Nurse Case Managers’ who are trained to refer patients to mental health services in addition to providing information on MG | 26% | 25% | 27% | 29% | 31% | 14% | 30% | 27% | 40% | 17% | 30% | 25% |
| Periodic updates of the latest information, developments and resources to talk about the unique ways MG can affect your life | 24% | 33% | 19% | 12% | 23% | 43% | 22% | 27% | 33% | 17% | 20% | 25% |
| Discussion guide for people living with MG to discuss treatment options with their doctor | 21% | 25% | 19% | 35% | 15% | 0% | 13% | 36% | 13% | 26% | 10% | 25% |
| Packets or brochures with MG treatment information | 18% | 25% | 15% | 6% | 31% | 29% | 9% | 36% | 20% | 17% | 20% | 18% |
| Transportation to the doctor’s office or treatment center | 18% | 8% | 23% | 12% | 23% | 29% | 17% | 18% | 13% | 22% | 0% | 25% |
| Social Worker support to manage ongoing challenges living with MG | 18% | 8% | 23% | 18% | 23% | 14% | 22% | 18% | 20% | 17% | 20% | 18% |
| Resource to guide conversations with your employer about your MG diagnosis and rights regarding employment | 13% | 8% | 15% | 12% | 15% | 14% | 17% | 0% | 13% | 13% | 30% | 7% |
| Lodging (eg, a place to stay for individuals living with MG and their caregivers) | 8% | 17% | 4% | 12% | 8% | 0% | 13% | 0% | 13% | 4% | 0% | 11% |
| None of the above | 3% | 8% | 0% | 6% | 0% | 0% | 0% | 9% | 0% | 4% | 0% | 4% |
| Other | 0% | 0% | 0% | 0% | 0% | 0% | 0% | 0% | 0% | 0% | 0% | 0% |
| n | 38 | 12 | 26 | 17 | 13 | 7 | 23 | 11 | 15 | 23 | 10 | 28 |

1. Continuing treatment

|  | Overall | Area of Residence | | Insurance Status | | | Education Level | | Ethnicity | | Employment Status | |
| --- | --- | --- | --- | --- | --- | --- | --- | --- | --- | --- | --- | --- |
|  |  | Rural or Small town | Urban or Suburban | Medicare | Medicaid | Private | Post-secondary | High school or GED | White/Caucasian | Non-White/Caucasian | Employed | Not Employed |
| Quick emergency “takeaway” resources for MG patients to carry/bring during a crisis or episode (eg, in the ER) | 45% | 42% | 46% | 47% | 62% | 14% | 48% | 45% | 40% | 48% | 20% | 54% |
| Collection of resources providing tips for practicing better physical and mental health in daily life with MG | 42% | 42% | 42% | 35% | 46% | 43% | 52% | 27% | 53% | 35% | 60% | 36% |
| Social community for individuals living with MG to share about their day-to-day experiences | 34% | 25% | 38% | 24% | 31% | 57% | 39% | 36% | 33% | 35% | 50% | 29% |
| ‘Nurse Case Manager’ to help individuals living with MG provide guidance switching to a new treatment (eg, insurance coverage, side effects, travel and logistics) | 34% | 25% | 38% | 47% | 15% | 43% | 39% | 9% | 40% | 30% | 40% | 32% |
| Tool to help individuals living with MG track symptoms | 34% | 17% | 42% | 24% | 38% | 57% | 39% | 18% | 33% | 35% | 60% | 25% |
| Social worker support to manage ongoing challenges living with MG | 26% | 25% | 27% | 12% | 31% | 57% | 39% | 9% | 27% | 26% | 20% | 29% |
| Forum where stories of people living with MG can be shared widely with others living with MG | 13% | 17% | 12% | 18% | 8% | 0% | 4% | 27% | 20% | 9% | 0% | 18% |
| Resource guide on the steps to take to find a qualified neurologist who can treat MG | 13% | 8% | 15% | 12% | 23% | 0% | 9% | 9% | 7% | 17% | 10% | 14% |
| Tool to search for nearby infusion centers for treatment using your zip-code | 11% | 8% | 12% | 18% | 0% | 14% | 9% | 18% | 13% | 9% | 20% | 7% |
| Discussion series for learning and sharing between MG patients and caregivers on how to manage living with MG | 11% | 25% | 4% | 6% | 15% | 14% | 9% | 18% | 13% | 9% | 0% | 14% |
| Discussion guides for how to talk to family and close friends about MG | 8% | 25% | 0% | 6% | 15% | 0% | 4% | 18% | 13% | 4% | 0% | 11% |
| None of the above | 5% | 8% | 4% | 12% | 0% | 0% | 0% | 18% | 0% | 9% | 0% | 7% |
| Transportation to the doctor’s office or treatment center | 5% | 8% | 4% | 6% | 8% | 0% | 4% | 9% | 7% | 4% | 10% | 4% |
| Other | 3% | 8% | 0% | 6% | 0% | 0% | 0% | 0% | 0% | 4% | 0% | 4% |
| MG-friendly cookbook with recipes, diet and meal-prep tips | 3% | 0% | 4% | 6% | 0% | 0% | 4% | 0% | 0% | 4% | 0% | 4% |
| Resource to guide conversations with your employer about your MG diagnosis and rights regarding employment | 3% | 0% | 4% | 0% | 8% | 0% | 0% | 0% | 0% | 4% | 10% | 0% |
| n | 38 | 12 | 26 | 17 | 13 | 7 | 23 | 11 | 15 | 23 | 10 | 28 |

# Figure S5: Preferred source of information and support by phase (diagnosis, accessing treatment, initiating treatment, and continuing treatment)

A B

C D

Respondents were asked to select 2 sources from which they would have liked to receive the 3 resources they expressed as most valuable in each phase. Response choices were shown in a randomized order.
